# Non-reassuring foetal status and sleep problems in 1-year-old infants in the Japan Environment and Children’s Study: a cohort study

**DOI:** 10.1038/s41598-020-67856-z

**Published:** 2020-07-10

**Authors:** Kazushige Nakahara, Takehiro Michikawa, Seiichi Morokuma, Masanobu Ogawa, Kiyoko Kato, Masafumi Sanefuji, Eiji Shibata, Mayumi Tsuji, Masayuki Shimono, Toshihiro Kawamoto, Shouichi Ohga, Koichi Kusuhara, The Japan Environment and Children’s Study Group, Michihiro Kamijima, Shin Yamazaki, Yukihiro Ohya, Reiko Kishi, Nobuo Yaegashi, Koichi Hashimoto, Chisato Mori, Shuichi Ito, Zentaro Yamagata, Hidekuni Inadera, Takeo Nakayama, Hiroyasu Iso, Masayuki Shima, Youichi Kurozawa, Narufumi Suganuma, Takahiko Katoh

**Affiliations:** 1 Department of Obstetrics and Gynecology, Graduate School of Medical Sciences, Kyushu University, Fukuoka, Japan; 2 Department of Environmental and Occupational Health, School of Medicine, Toho University, Tokyo, Japan; 3 Department of Health Sciences, Graduate School of Medical Sciences, Kyushu University, Fukuoka, 812-8582 Japan; 4 Research Center for Environmental and Developmental Medical Sciences, Kyushu University, Fukuoka, Japan; 5 Department of Pediatrics, Graduate School of Medical Sciences, Kyushu University, Fukuoka, Japan; 6 Japan Environment and Children’s Study, UOEH Subunit Center, University of Occupational and Environmental Health, Kitakyushu, Fukuoka Japan; 7 Department of Obstetrics and Gynecology, School of Medicine, University of Occupational and Environmental Health, Kitakyushu, Fukuoka Japan; 8 Department of Environmental Health, School of Medicine, University of Occupational and Environmental Health, Kitakyushu, Fukuoka Japan; 9 Department of Pediatrics, School of Medicine, University of Occupational and Environmental Health, Kitakyushu, Japan; 10 Nagoya City University, Nagoya, Japan; 11 National Institute for Environmental Studies, Tsukuba, Japan; 12 National Center for Child Health and Development, Tokyo, Japan; 13 Hokkaido University, Sapporo, Japan; 14 Tohoku University, Sendai, Japan; 15 Fukushima Medical University, Fukushima, Japan; 16 Chiba University, Chiba, Japan; 17 Yokohama City University, Yokohama, Japan; 18 University of Yamanashi, Chuo, Japan; 19 University of Toyama, Toyama, Japan; 20 Kyoto University, Kyoto, Japan; 21 Osaka University, Suita, Japan; 22 Hyogo College of Medicine, Nishinomiya, Japan; 23 Tottori University, Yonago, Japan; 24 Kochi University, Nankoku, Japan; 25 Kumamoto University, Kumamoto, Japan

**Keywords:** Medical research, Epidemiology, Risk factors

## Abstract

Abnormal autonomic function may cause false-positive non-reassuring foetal status (*fp*NRFS) and may also cause sleeping problems after birth. However, an association between *fp*NRFS and sleeping problems in infants has not been reported. We previously showed an association of NRFS with temperament, including bad mood and frequent crying for long durations in 1-month-old infants. In the present study, we aimed to assess this association in 1-year-old infants. A total of 62,612 single pregnant women were included in the analysis. *fp*NRFS was identified from medical records. Sleep problems, such as short sleep duration or crying at night, were investigated in 1-year-old infants using a questionnaire for mothers. We used a log-binominal regression model to explore the association of *fp*NRFS with each sleep problem and to estimate risk ratios (RRs). The number of *fp*NRFS cases was 2,071, with a frequency of 3.3%. We observed an association of *fp*NRFS with shorter sleep duration of less than 8 h a night (RR 1.30, 95% confidence intervals [CI] 1.10–1.54), crying at night (RR 1.19, 95% CI 1.03–1.39), and bedtime after 22:00 (RR 1.09, 95% CI 1.00–1.18). *fp*NRFS may be associated with sleep problems in 1-year-old infants.

## Introduction

Children with developmental disorders tend to have sleep problems and abnormal temperaments^1,2,3,4^. Foetal distress is one of the prenatal risk factors for developmental disorders, such as autism spectrum disorder (ASD)^5^.

No non-invasive methods have been developed to determine actual foetal distress with foetal acidosis. Obstetricians clinically use non-reassuring foetal status (NRFS) by monitoring foetal heart rate using cardiotocography (CTG) instead of foetal distress. Unfortunately, the false-positive rate of NRFS assessment by CTG is high^6–8^. Many diagnosed NRFS cases do not show actual foetal distress. They demonstrate normal umbilical cord blood pH and normal Apgar scores.

Babies with normal umbilical cord blood and normal Apgar scores may be categorised into two groups. Some babies show abnormal heart rate patterns and are diagnosed with false-positive NRFS (*fp*NRFS), whereas others do not. Babies showing *fp*NRFS may inherently have abnormal regulation of heart rate. Autonomic nerves play an important role in heart rate regulation^9^ and are also closely related to sleep^10^. Children with ASD tend to have different heart rate variability from that of normal children^11^, and they frequently have sleep disorders, such as short sleep during the night and frequent night crying^12,13^. Therefore, children with *fp*NRFS may have abnormal autonomic functions and could show abnormal sleep patterns after birth.

We previously showed an association of NRFS with temperaments, including bad mood and frequent crying for a long duration in 1-month infants^14^. These findings suggest that NRFS may be associated with sleep disorders; nevertheless, an association between the two has not been reported. Therefore, this study aimed to investigate the presence of an association between NRFS, especially *fp*NRFS, and sleep problems in 1-year-old infants.

## Methods

Data used in this study were obtained from the Japan Environment and Children’s Study (JECS), an ongoing large-scale cohort study. The JECS was designed to follow-up children from the prenatal period to the age of 13 years. The baseline profile of participants in the JECS was reported previously^15^.

### Ethics of research

The JECS study protocol was approved by the Ministry of the Environment’s Institutional Review Board on Epidemiological Studies (No. 100406001) and the Ethics Committee of all participating institutions: the National Institute for Environmental Studies that leads the JECS, the National Center for Child Health and Development, Hokkaido University, Sapporo Medical University, Asahikawa Medical College, Japanese Red Cross Hokkaido College of Nursing, Tohoku University, Fukushima Medical University, Chiba University, Yokohama City University, University of Yamanashi, Shinshu University, University of Toyama, Nagoya City University, Kyoto University, Doshisha University, Osaka University, Osaka Medical Center and Research Institute for Maternal and Child Health, Hyogo College of Medicine, Tottori University, Kochi University, University of Occupational and Environmental Health, Kyushu University, Kumamoto University, University of Miyazaki, and University of Ryukyu. Written informed consent was obtained from all participants. All methods were performed in accordance with approved guidelines. The detailed protocol has been reported elsewhere^16^.

### Study participants

**Figure 1:**
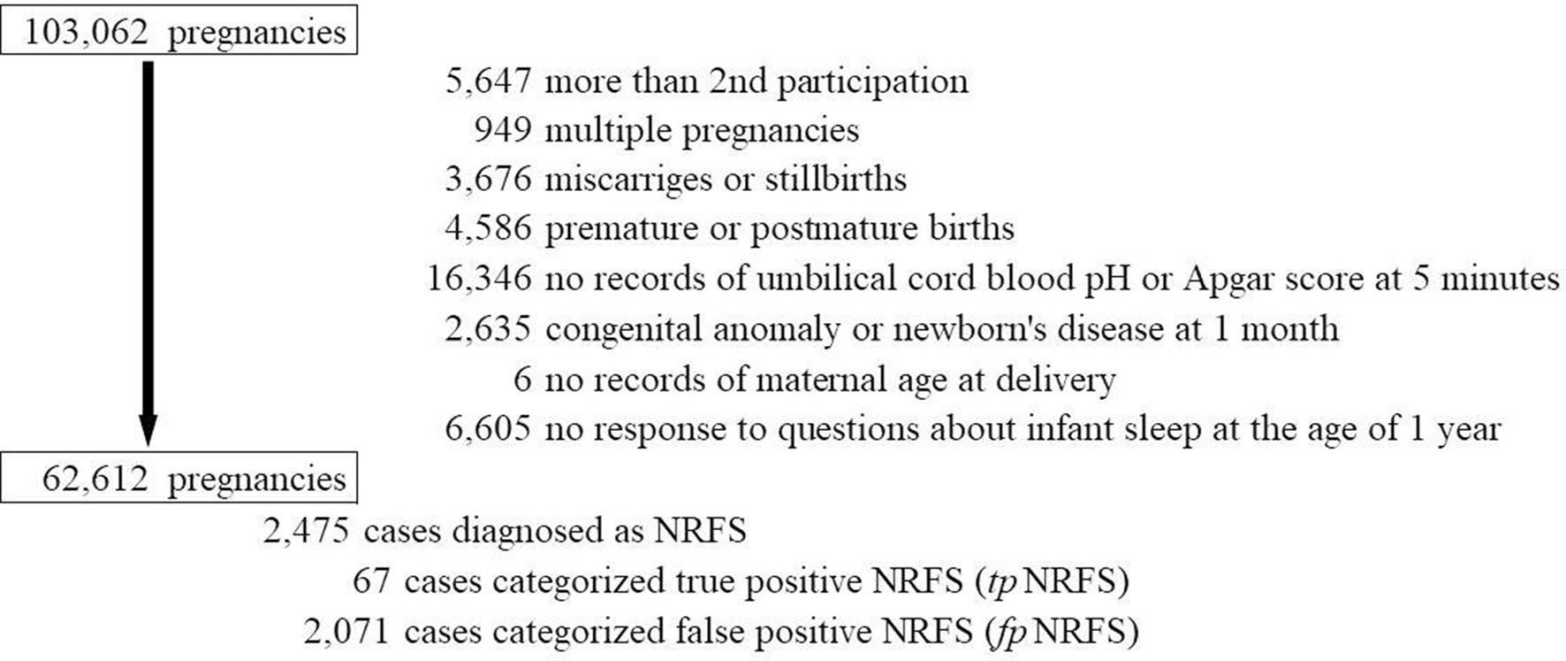
Population flow chart. NRFS: non-reassuring foetal status; true-positive NRFS (*tp*NRFS): cases with umbilical cord pH < 7.2 and Apgar score of < 7 at 5 min after birth of all NRFS cases; false-positive NRFS (*fp*NRFS): cases with umbilical cord pH ≥ 7.2 and Apgar score of ≥ 7 at 5 min after birth of all NRFS cases.

Between 2011 and 2014, 103,062 pregnancies were registered from 15 regions throughout Japan (Fig. 1). Of those, we excluded 40,450 pregnancies due to the following reasons: prior participation in the study (n = 5,647), multiple pregnancies (n = 949), miscarriage or stillbirth (n = 3,676), pre-term or post-term birth (n = 4,586), no records of umbilical cord blood pH or Apgar score at 5 min (n = 16,346), congenital anomaly or newborn disease at 1 month old (n = 2,635), missing information on maternal age at delivery (n = 6), and no response to the questions about infant sleep at the age of 1 year (n = 6,605). Finally, 62,612 single pregnant women were included in our analysis.

### Exposure (NRFS)

In Japan, abnormal patterns in foetal heart rate are categorised into five levels based on baseline heart rate, variability, kinds, severity of deceleration, and so on^17^. Level 1 is considered normal, while levels 3 or higher are usually diagnosed as NRFS, and require preparation and execution of forced delivery.

In the present study, the NRFS cases were selected in two ways, based on the medical record transcription at birth. First, we selected cases diagnosed as NRFS by obstetricians. In the cohort data, only information on the presence or absence of NRFS was available. Second, we selected cases that were not diagnosed as NRFS but showed umbilical cord blood pH < 7.2 and Apgar scores < 7 at 5 min after birth.

Of all the NRFS cases, those showing umbilical cord blood pH < 7.2 and Apgar scores of < 7 at 5 min were categorised as true-positive NRFS (*tp*NRFS) cases, and those showing umbilical cord blood pH ≥ 7.2 and Apgar scores ≥ 7 at 5 min were categorised as false-positive NRFS (*fp*NRFS) cases. Only cases that met either umbilical cord blood pH or Apgar scores at 5 min were excluded from the stratified analysis with *tp*NRFS and *fp*NRFS.

### Outcome (infant sleep and crying at night)

At 1 year after delivery, information on infant sleep and crying at night was collected via a parent-reported questionnaire. In this analysis, we focused on five points. First, from the responses regarding infant’s sleeping period the previous day, we determined the number of nocturnal awakenings. We defined ≥ 3 awakenings as too many because a previous study reported that the upper limit of number of awakenings during the night is 2.5 for 1-year-old infants.^18^ Second, we analysed whether the infants awoke more than once and kept awake for more than 1 h during the night. Third, we analysed the duration of sleep during the night (20:00–7:59). We regarded less than 8 h as unusual. Fourth, we determined bedtime of infants. We defined bedtime after 22:00 as too late. Fifth, we analysed crying at night in the past month. If the mother answered that her infant awoke and cried during the night and that the frequency of crying at night was more than 5 times in a week, we defined the case as crying at night.

### Covariates

Information about maternal age at delivery, smoking habits, alcohol consumption, pre-pregnancy body mass index (BMI), parity, gestational age at birth, infertility treatment, type of delivery, small for gestational age, infant sex, maternal psychological distress at 1 year after delivery, physician diagnosis of asthma and atopic dermatitis at 1 year old, and feeding status were collected via self-administered questionnaires and/or medical records. Maternal psychological distress was assessed using the Kessler 6^19,20^, including the questionnaire at 1 year after delivery. According to previous studies, participants with scores of 5 or more were categorised as having distress^21^.

### Statistical analyses

We used a log-binominal regression model to explore the association of NRFS with each outcome and to estimate the risk ratio (RRs) of each outcome and 95% confidence intervals (CIs). We initially adjusted for maternal age at delivery and then further adjusted for smoking habits (never smokers, ex-smokers who quit before pregnancy, smokers during early pregnancy), alcohol consumption (never drinkers, ex-drinkers who quit before pregnancy, drinkers during early pregnancy), pre-pregnancy BMI (< 18.5, 18.5–24.9, ≥ 25.0 kg/m^2^), parity (0, ≥ 1), infertility treatment (no, ovulation stimulation/artificial insemination by sperm from husband, assisted reproductive technology), type of delivery (vaginal, caesarean section), gestational age at birth (37–38, 39–41 weeks), small for gestational age (yes, no), psychological distress at 1 year after delivery (yes, no), doctor diagnosis of asthma and atopic dermatitis at 1 year old, and feeding (breast milk, synthetic milk, both).

We used a fixed dataset “jecs-an-20180131,” which was released in March 2018. Stata version 15 (StataCorp LP, College Station, Texas, USA) was used for all the analyses.

## Results

The baseline characteristics of the present study population with or without NRFS are shown in Table 1. The number of all NRFS cases was 2,475, with a frequency of 4.0%. Among the NRFS cases, 67 cases (2.7%) were *tp*NRFS, 2,071 cases (83.7%) were *fp*NRFS. The other 337 cases (13.7%) did not belong to any groups because these cases met only either umbilical cord pH or Apgar scores at 5 min.

**Table 1:** Baseline characteristics of the study population.

	Without non-reassuring foetal status (n = 60,137)		With non-reassuring foetal status (n = 2,475)	
	n^a^	%	n^a^	%
**Maternal characteristics**				
Age at delivery (years)				
< 25	5,437	9.0	202	8.2
25–29	16,536	27.5	654	26.4
30–34	21,513	35.8	887	35.8
≥ 35	16,651	27.7	732	29.6
Smoking habits				
Never smoked	35,745	59.6	1,512	61.3
Ex-smokers who quit before pregnancy	14,175	23.7	505	20.5
Smokers during early pregnancy	10,028	16.7	451	18.3
Alcohol consumption				
Never drank	20,847	34.7	804	32.6
Ex-drinkers who quit before pregnancy	11,001	18.3	401	16.2
Drinkers during early pregnancy	28,161	46.9	1,265	51.2
Pre-pregnancy body mass index, kg/m^2^				
< 18.5	9,613	16.0	401	16.2
18.5–24.9	44,463	74.0	1,803	72.9
≥ 25.0	6,041	10.1	270	10.9
Parity				
0	25,965	43.3	1,874	75.9
≥ 1	33,997	56.7	594	24.1
Infertility treatment				
No	56,104	93.3	2,173	87.9
Ovulation stimulation/artificial insemination by sperm from husband	2,203	3.7	139	5.6
Assisted reproductive technology	1,798	3.0	161	6.5
Type of delivery				
Vaginal	50,555	84.1	1,201	48.5
Caesarean	9,532	15.9	1,274	51.5
Gestational age (weeks)				
Early term (37–38)	19,779	32.9	558	22.6
Full term (39–41)	40,358	67.1	1,917	77.5
Educational background (years)				
< 10	2,428	4.1	92	3.8
10–12	18,404	30.9	683	27.8
13–16	37,813	63.5	1,641	66.9
≥ 17	876	1.5	37	1.5
Household income (million Japanese-yen/year)				
< 2	2,908	5.2	119	5.2
2 to < 4	18,989	34.1	747	32.4
4 to < 6	18,531	33.3	748	32.5
6 to < 8	9,060	16.3	412	17.9
8 to < 10	3,769	6.8	181	7.9
≥ 10	2,399	4.3	97	4.2
Kessler six-item psychological distress scale at 1 year				
0–4	46,845	78.0	1,983	80.3
> = 5 (psychological distress)	13,183	22.0	487	19.7
Birth weight				
Mean (SD) (g)	3.065 (352)		2,987 (407)	
Small for gestation age	4,121	6.9	395	16.0
Infant sex				
Male	30,418	50.6	1,429	57.7
Female	29,719	49.4	1,046	42.3
**Infant characteristics**				
Doctor diagnosis at 1 year old				
Asthma	1,528	2.5	38	1.5
Atopic dermatitis	2,588	4.3	105	4.2
Feeding status				
Formula feeding	1,298	2.2	47	1.9
Partial breastfeeding	38,258	63.6	1,758	71.0
Exclusive breastfeeding	20,581	34.2	670	27.1

^a^Numbers in subgroups do not equal the overall number because of missing data.

Table 2 shows the RRs for NRFS and infant sleep and crying at night. In the multivariable model, we observed the association of all NRFS with shorter sleep time less than 8 h during the night (RR 1.28, 95% CI 1.10–1.49), crying at night (RR 1.17, 95% CI 1.02–1.34) and bedtime after 22:00 (RR 1.10, 95% CI 1.01–1.18). The same associations were also observed only in the *fp*NRFS cases (RR for short sleep 1.30, 95% CI 1.10–1.54, RR for crying at night = 1.19, 95% CI 1.03–1.39, RR for bedtime after 22:00 = 1.09, 95% CI 1.00–1.18). *tp*NRFS was not associated with any outcomes.

**Table 2:** Association between non-reassuring foetal status and infantile sleep at 1 year of age.

	n	Number of outcomes	Frequency of outcome (%)	Maternal age adjusted model			Multivariable model^a^		
				RR	95% CI		RR	95% CI	
**All NRFS**									
Waking up 3 or more times in a night									
No NRFS	59,697	1,426	2.4	**Ref**			**Ref**		
NRFS	2,456	67	2.7	**1.14**	0.89	1.45	**1.20**	0.93	1.55
Waking up 1 or more times and remaining awake for more than 1 h									
No NRFS	59,697	3,435	5.8	**Ref**			**Ref**		
NRFS	2,456	153	6.2	**1.08**	0.93	1.27	**1.01**	0.86	1.19
Sleep for less than 8 h during the night (20:00–7:59)									
No NRFS	59,697	3,067	5.1	**Ref**			**Ref**		
NRFS	2,456	174	7.1	**1.37**	1.19	1.59	**1.28**	1.10	1.49
Sleep at 22:00 or later									
No NRFS	59,697	11,929	20.0	**Ref**			**Ref**		
NRFS	2,456	573	23.3	**1.17**	1.09	1.26	**1.10**	1.01	1.18
Crying for 5 days or over in a week									
No NRFS	60,054	4,452	7.4	**Ref**			**Ref**		
NRFS	2,474	207	8.4	**1.13**	0.99	1.29	**1.17**	1.02	1.34
**True-positive NRFS (tpNRFS)**									
Waking up 3 or more times in a night									
No NRFS	59,697	1,426	2.4	**Ref**			**Ref**		
NRFS	66	3	4.6	**1.95**	0.64	5.88	**2.08**	0.69	6.26
Waking up 1 or more times and remaining awake for more than 1 h									
No NRFS	59,697	3,435	5.8	**Ref**			**Ref**		
NRFS	66	6	9.1	**1.56**	0.73	3.36	**1.36**	0.63	2.91
Sleep for less than 8 h during the night (20:00–7:59)									
No NRFS	59,697	3,067	5.1	**Ref**			**Ref**		
NRFS	66	2	3.0	**0.59**	0.15	2.29	**0.50**	0.13	1.95
Sleep at 22:00 or later									
No NRFS	59,697	2,409	4.0	**Ref**			**Ref**		
NRFS	66	12	18.2	**0.92**	0.55	1.53	**0.83**	0.50	1.38
Crying for 5 days or over in a week									
No NRFS	60,054	4,452	7.4	**Ref**			**Ref**		
NRFS	67	8	11.9	**1.63**	0.85	3.12	**1.64**	0.86	3.12
**False-positive NRFS (fpNRFS)**									
Waking up 3 or more times in a night									
No NRFS	59,697	1,426	2.4	**Ref**			**Ref**		
NRFS	2,055	60	2.9	**1.21**	0.94	1.57	**1.28**	0.98	1.68
Waking up 1 or more times and remaining awake for more than 1 h									
No NRFS	59,697	3,435	5.8	**Ref**			**Ref**		
NRFS	2,055	131	6.4	**1.11**	0.94	1.31	**1.04**	0.87	1.23
Sleep for less than 8 h during the night (20:00–7:59)									
No NRFS	59,697	3,067	5.1	**Ref**			**Ref**		
NRFS	2,055	148	7.2	**1.40**	1.19	1.64	**1.30**	1.10	1.54
Sleep at 22:00 or later									
No NRFS	59,697	11,929	20.0	**Ref**			**Ref**		
NRFS	2,055	474	23.2	**1.16**	1.07	1.26	**1.09**	1.00	1.18
Crying at night for 5 days or over in a week									
No NRFS	60,054	4,452	7.4	**Ref**			**Ref**		
NRFS	2,071	176	8.5	**1.14**	0.99	1.32	**1.19**	1.03	1.39

*CI* confidence interval, *RR* risk ratio.

^a^Adjusted for maternal age at delivery, smoking habits, alcohol consumption, pre-pregnancy body mass index, parity, infertility treatment, type of delivery, gestational age at birth, small for gestational age, infant sex, psychological distress at 1 year after delivery, doctor diagnosis of asthma and atopic dermatitis at 1 year old, and feeding status.

## Discussion

This study showed that children with NRFS before birth tended to have sleep problems at 1 year old. The association of *fp*NRFS with sleep problems was similar to that of all NRFS with sleep problems. This finding was attributed to children with *fp*NRFS comprising most of the group with all NRFS. *tp*NRFS was not associated with any outcomes; however, the number of *tp*NRFS cases was few, limiting the ability to make conclusions.

The present study had two major limitations. First, there could be unmeasured confounding factors, such as parental life rhythm and other siblings. Second, child sleep was evaluated using a questionnaire for mothers, which might introduce some bias. On the other hand, a strong point of the present study was the large sample size collected nationwide.

We previously reported an association between NRFS and temperament at 1 month after birth^14^. The present study showed that NRFS before birth appeared to influence sleep at 1 year. The possible reason for this is that temperament and sleep are formed prenatally^22,23^, and foetal temperament affects heart rate reaction to stress.

It is conceivable that children with sleep problems in early infancy are more likely to have developmental disorders than those without sleep problems. Thus, careful follow up of neonates showing NRFS may lead to early detection of developmental disorders.

On the other hand, the RRs for sleeping problems in the NRFS group for sleep problems were not very large. Further investigations are needed to confirm an association between NRFS and sleeping problems in infants.

In conclusion, *fp*NRFS may be associated with sleep problems in 1-year-old infants.

### Ethical approval

The study protocol was approved by the Ministry of the Environment’s Institutional Review Board on Epidemiological Studies (No. 100406001) and the Ethics Committee of all participating institutions. Written informed consent was obtained from all participants.
